# Demographic and Diagnostic Factors in Physical Therapy Attendance

**DOI:** 10.7759/cureus.55908

**Published:** 2024-03-10

**Authors:** William Leatherwood, Adrian Torres, Sofia Hidalgo Perea, Megan Paulus

**Affiliations:** 1 Department of Orthopaedic Surgery, Stony Brook University, New York, USA

**Keywords:** sports medicine, orthopedic rehabilitation, orthopedics, physical therapy attendance, physical therapy

## Abstract

Introduction

Physical therapy (PT) is an effective nonoperative treatment for various orthopedic diagnoses. However, patients may have many reasons to dismiss PT, including favoring another intervention for their injury, time constraints, transportation, and cost. This dismissal of PT may contribute to inadequate patient compliance. This study aimed to elucidate patient compliance with a basic PT prescription and whether PT led to subjective injury improvement.

Methods

This is a retrospective study of patients observed in Stony Brook Orthopedic clinics from 08/01/2022 to 12/23/2022. Patients prescribed PT received a phone call six weeks after the PT prescription. The primary outcome was patient attendance at PT. Secondary outcomes were subjective; symptomatic improvement was listed as better, worse, or the same. Chi-square testing was used to compare outcomes.

Results

A total of 100 patients were enrolled in the study. Patients prescribed PT following surgery were more likely to attend compared to patients prescribed PT as a primary treatment (P value=0.027). The association between attendance at PT and a change in subjective symptoms (better, worse, same) was not significant. Patients’ age, sex, and chronicity of injury were not significant factors in PT attendance. Of the 40 patients who did not attend PT, 14 cited time constraints, 11 utilized self-directed treatment, three cited insurance, two cited transportation, and 10 cited other reasons.

Conclusions

Overall, postoperative patients were more likely to attend PT compared to patients prescribed PT as a primary treatment. Factors such as age, sex, and chronicity of injury did not affect whether a patient attended PT. Of the patients enrolled, 71% stated subjective improvement in symptoms, but there was no association between symptoms and PT attendance. This study highlights the characteristics of those patient factors that may influence PT compliance and underscores the importance of further research into the population most likely to attend and benefit from PT.

## Introduction

Physical therapy (PT) is an established nonoperative method for treating a range of acute and chronic musculoskeletal conditions. It is often used as a primary treatment and has demonstrated success in preventing and treating conditions such as subacromial shoulder pain, frozen shoulder, rotator cuff tendinopathy, rotator cuff tears, ankle sprains, knee osteoarthritis, and more [1-8]. Studies have also demonstrated that pursuing nonoperative treatment, such as with PT, has comparable outcomes regarding function, pain, and range of motion when compared to operative treatment in conditions such as rotator cuff injuries [1]. These previous studies underscore the importance of consistent PT attendance and utilization as prescribed, as PT recommendations can aid in avoiding invasive surgeries while maintaining similar outcomes.

As a primary form of treatment, PT has also been demonstrated as a cost-effective intervention in patients suffering from carpal tunnel syndrome, sharing similar improvement in function when compared to surgical treatment while accruing fewer expenses [9]. In addition to limiting healthcare costs, PT can also offset the risks, complications, and adverse outcomes associated with undergoing an operative procedure. Additionally, in patients who undergo surgery, PT is often used as an adjunct postoperatively and is believed to be crucial to the long-term success of many operations. This may be through optimizing surgical results and improving patients' subjective views of their disability and behavior [10-13]. When discussing PT, patients can be indifferent and look for different means to achieve reduced pain or improved functionality, especially when prescribed longer PT durations or if the patient has low activity at baseline. This skepticism or belief of increased pain because of PT may manifest with nonattendance when it is prescribed [14,15].

When prescribing PT, a six-week course is typically pursued for various orthopedic injuries. This six-week time course has been demonstrated to have improved outcomes from baseline while providing similar outcomes to longer PT durations [16]. However, in an office setting, it may be challenging to determine patient adherence to the prescribed regimen and the number of sessions a patient must attend before benefits are appreciated. Moreover, many variables may be associated with being unable to participate in PT, such as job restrictions, transportation, insurance, family life, patients' sentiments about PT, or decisions to pursue a home exercise program. As previous studies have shown PT to be successful for many general orthopedic diagnoses, it would be particularly valuable to know the precise compliance rate of PT when prescribed [1-13]. This information could aid clinicians in guiding patient discussions and treatment.

While data on PT treatment of specific diagnoses are abundant, data on patient compliance with prescribed, supervised PT and the variables associated with PT attendance are very limited [1-13]. Therefore, this study aimed to further evaluate patient adherence when prescribed a basic PT regimen for various orthopedic diagnoses. This study further specified the subjective nature of patients' injuries after PT prescription and patient characteristics associated with PT prescription attendance.

## Materials and methods

After Institutional Review Board approval, patients seen in Stony Brook Orthopedics outpatient clinics between August 2022 and December 2022 were retrospectively reviewed. Clinical schedules of two sports medicine providers, one hand provider, and one foot and ankle provider were sequentially evaluated, and patients who were prescribed a supervised PT regimen for either primary or postoperative treatment were identified. Patient diagnoses, chronicity of the injury, and whether PT was for primary or postoperative treatment were recorded. Patients under eighteen years old, prisoners, or cognitively impaired were excluded. Patients received a phone call six weeks after being prescribed PT and answered a standard questionnaire after verbal consent to participate was received. Information collected included whether they attended PT, the number of sessions, the reason for not attending, and if the injury for which they were prescribed PT was subjectively better, worse, or the same. This process was continued until data for 100 patients were collected, as a similar number of subjects has demonstrated sufficient power in other orthopedic studies [17,18]. A total of 204 patients met the inclusion criteria and received phone calls six weeks after receiving a PT prescription. Patients were further excluded if they could not be reached by phone call within three phone calls, declined to participate at the time of the phone call, were found to have had PT prescribed for their current diagnosis for longer than six weeks, or if a secondary outcome of subjective symptoms could not be elicited. A total of 100 patients were included in the final analysis, consistent with previous phone survey studies [19].

Analyses were conducted using Statistical Product and Service Solutions (SPSS, version 22.0; IBM SPSS Statistics for Windows, Armonk, NY). Chi-square testing was used to compare outcomes. Descriptive data included age, sex, provider subspeciality, chronicity of injury, primary or postoperative PT, whether they attended PT, number of sessions, and reasons for not attending PT. Chi-square testing included comparing subjective improvement to PT attendance, sex, age, chronicity of injury, primary or postoperative treatment, and comparing attendance to sex, age, chronicity of injury, and primary or postoperative PT. We then subdivided those who attended PT and those who did not and completed the same comparisons.

## Results

**Figure 1:**
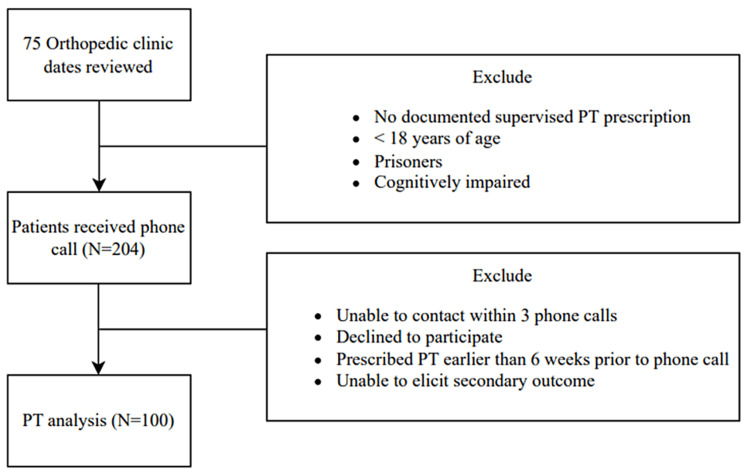
Consort diagram depicting patient enrollment.

The final analysis included 100 patients who were prescribed PT for either primary or postoperative treatment (Figure 1). As demonstrated in Table 1, the patient descriptive characteristics showed an average age of 49.8 +/-15.8 (range: 19-85), with 46 males and 54 females. Forty-five of the PT prescriptions were for acute injuries, 55 were for chronic injuries, 79 were for primary treatment of an injury, and 21 were for postoperative treatment. The orthopedic subspecialty providing PT prescriptions comprised six from hand providers, 64 from sports providers, and 30 from foot and ankle providers. Forty of the patients prescribed PT did not attend, while 60 attended for at least one session. Patients who attended PT averaged 11.75 +/- 6.5 sessions (range: 1-36), with primary treatment patients averaging 10.4 visits and postoperative patients averaging 15.1 visits. Of those who attended PT, patients who did not attend PT cited time constraints and the use of self-directed home therapy as the top reasons for not attending.

**Table 1:** Patient descriptive characteristics included in analysis. PT: physical therapy; Tx: treatment

Variable	Number of patients
Average age (years)*	49.8 ± 15.81
Range (years)	19-85
Sex	
Male	46
Female	54
Provider	
Hand	6
Sports	12
Foot and ankle	30
Sports	52
Type of injury	
Acute	45
Chronic	55
Type of PT	
Primary Tx	79
Post-op Tx	21
Did they attend PT?	
No	40
Yes	60
If Yes, how many PT sessions?*	11.75 ± 6.5
Range of PT sessions	1-36
If No, why?	
Co-pay/insurance	3
Self-directed	11
Time constraints	14
Transportation	2
Other	10
*Mean ± SD, range	

As shown in Table 2, 71 of the 100 patients reported subjective improvement in their symptoms, with 44 of those attending PT. Twenty-two total patients reported they subjectively felt the same. In contrast, seven reported that they were subjectively worse than before PT. This shows a slightly increasing trend of subjective improvement of those attending PT, although it did not reach statistical significance. No differences were observed in attendance or subjective improvement related to sex, age, or chronicity of injury. However, there was a significant difference in patients attending PT for postoperative PT compared to PT for primary treatment (P value 0.027) (Table 3). However, there was no difference in subjective improvement in patients who attended PT for primary or postoperative treatment (Table 4).

**Table 2:** Patient’s subjective improvement following attending or not attending PT.

Subjective improvement	Overall	Yes	No
Better	71	44	27
Same	22	12	10
Worse	7	4	3

**Table 3:** Comparison of operative status, injury characteristics, and patient demographic factors and attendance to PT.

Variable	Yes	No	P value
Operative status			
Postoperative	17	4	0.027
Primary treatment	43	36	
Sex			
Male	27	19	0.806
Female	33	21	
Age			
65+	10	5	0.5674
Less than 65	50	35	
Injury chronicity			
Acute injury	29	16	0.412
Chronic injury	31	24	

**Table 4:** Comparison of operative status, injury characteristics, patient demographic factors, and final injury outcome at the time of the phone call.

Variable	Better	Same	Worse	P value
Operative status				
Postoperative	14	3	0	0.385
Primary treatment	30	9	4	
Sex				
Male	19	6	2	0.896
Female	25	6	2	
Age				
65+	9	1	0	0.3955
Less than 65	35	11	4	
Injury chronicity				
Acute injury	23	5	1	0.507
Chronic injury	21	7	3	

## Discussion

Many studies have demonstrated the benefits of PT in the recovery of musculoskeletal injuries and the influence of PT attendance on postoperative outcomes [1-8,11-13]. Nevertheless, patient adherence to the prescribed plan remains an issue for many clinicians, with one study finding that only 42.7% completed the recommended PT plan [20].

These results expand upon the existing literature and document novel patient population factors associated with adherence to a prescribed PT plan. There is a clear demonstration that postoperative patients prescribed PT were more likely to attend compared to those prescribed PT as a primary treatment; however, factors such as sex, age, and chronicity had no association with whether a patient attended PT. Of those patients enrolled, 71 reported subjective improvement in symptoms in the six weeks from the initial PT prescription to the phone call they received. However, no significant improvement in subjective symptoms was observed between the population who attended PT and those who did not. Additionally, 40 of the patients at our institution did not attend the prescribed PT. This was a slightly lower nonattendance rate than that documented in previous studies [20]. Of the patients who attended PT, the average number of visits was 11.75, ranging from one to 36 visits. Interestingly, the patients attending PT for postoperative treatment attended nearly 50% more sessions than those for primary treatment. Several factors may have contributed to this finding, as patients prescribed PT for postoperative treatment may have had more severe functional deficits following surgery or different intrinsic motivators to attend PT to obtain a satisfactory surgical outcome. Postoperative patients may have also had more frequent and lengthier discussions with their surgeons on the importance of PT adherence to their surgery's success.

While this study did not find an association between subjective improvement in injury and PT attendance, this finding could be secondary to including all orthopedic diagnoses in our analysis. Specific orthopedic diagnoses demonstrate more objective benefits from PT attendance [3,13]. However, we chose to include all musculoskeletal diagnoses to reflect the heterogeneity in the population of many orthopedic offices. Our study evaluated a basic PT prescription for supervised PT, whereas a specific injury, guided by a distinct treatment or targeted outcome, may have given physical therapists more direct guidance and patients more motivation to obtain optimal results. This study also considers PT as supervised rehabilitation. We assessed whether home exercises were a factor in non-attendance to supervised PT, but we did not evaluate if all non-attending patients were conducting home exercises. Other limitations to our study include reliance on patient recall to PT attendance and the patient's impression of their injury resolution rather than an objective scale on functionality.

PT is an essential nonsurgical treatment modality in the physician's arsenal, and successful treatment with PT likely begins in the office with the initial physician-patient discussion. If thorough explanations and emphasis are placed on the patient's diagnosis and the positive effects PT can have on its resolution, then patients may be more inclined and motivated to attend and adhere to PT. Additionally, it is also essential to set expectations on what the patient may achieve with PT and the time frame it may take to reach the final results, while also considering psychosocial factors in the discussion, as this has also been shown to affect PT results [21].

## Conclusions

Patient factors, including age, gender, and chronicity of injury, were not associated with PT attendance or final injury outcome. The patients most likely to attend PT were those prescribed PT for postoperative therapy. The most cited reason for nonattendance was time constraints, followed by pursuing self-directed therapy at home. Transportation and costs associated with co-payment or insurance were the least cited reasons for nonattendance. Based on these findings, it is evident that patients prescribed PT for nonoperative pathologies may benefit from more thorough discussions with their physicians on its benefits.
